# Supplementary material for: Physical Activity Assessed by Wrist and Thigh Worn Accelerometry and Associations with Cardiometabolic Health
Source: Sensors (Basel). 2023 Aug 23;23(17):7353. doi: 10.3390/s23177353 (PMC10489920; doi:10.3390/s23177353)
Supplement: Supplementary file 1 [file sensors-23-07353-s001.zip › sensors-2456685-supplementary.pdf]

Supplementary Table S1. Association between Axivity- and activPAL-derived volume and intensity and cardiometabolic health.

	Axivity			ActivPAL		
**Health marker**	**Unadjusted**	**Model 1**	**Model 2**	**Unadjusted**	**Model 1**	**Model 2**
**Cardiometabolic risk score**						
Average acceleration (m*g*)	**-0.115 (-0.167 to -0.064)**	**-0.070 (-0.113 to -0.027)**	**-0.022 (-0.072 to 0.029)**	**-0.080 (-0.135 to -0.025)**	**-0.109 (-0.159 to -0.059)**	**-0.133 (-0.193 to -0.073)**
Intensity gradient	**-0.114 (-0.165 to -0.064)**	**-0.102 (-0.146 to -0.058)**	**-0.090 (-0.143 to -0.036)**	0.019 (-0.033 to 0.071)	-0.024 (-0.071 to 0.023)	0.045 (-0.011 to 0.101)
**Body mass index**						
Average acceleration (m*g*)	**-1.067 (-1.550 to -0.583)**	**-0.892 (-1.399 to -0.385)**	**-0.571 (-1.135 to -0.007)**	**-1.025 (-1.512 to -0.537)**	**-0.967 (-1.465 to -0.469)**	**-1.183 (-1.770 to -0.595)**
Intensity gradient	**-1.067 (-1.514 to -0.621)**	**-0.922 (-1.381 to -0.463)**	**-0.598 (-1.098 to -0.099)**	-0.142 (-0.574 to 0.290)	-0.212 (-0.653 to 0.229)	0.407 (-0.112 to 0.926)
**Waist circumference (cm)**						
Average acceleration (m*g*)	**-3.039 (-4.206 to -1.871)**	**-2.430 (-3.569 to -1.292)**	**-1.477 (-2.761 to -0.193)**	**-1.986 (-3.187 to -0.785)**	**-2.669 (-3.831 to -1.508)**	**-3.208 (-4.619 to -1.796)**
Intensity gradient	**-2.646 (-3.722 to -1.571)**	**-2.610 (-3.655 to -1.566)**	**-1.775 (-2.934 to -0.615)**	0.330 (-0.739 to 1.399)	-0.666 (-1.702 to 0.371)	1.013 (-0.241 to 2.266)
**Body fat (%)**						
Average acceleration (m*g*)	**-1.563 (-2.272 to -0.855)**	**-0.998 (-1.524 to -0.471)**	**-0.694 (-1.324 to -0.065)**	**-2.849 (-3.576 to -2.122)**	**-1.115 (-1.673 to -0.557)**	**-1.334 (-1.964 to -0.704)**
Intensity gradient	**-2.462 (-3.150 to -1.773)**	**-0.950 (-1.426 to -0.474)**	**-0.563 (-1.124 to -0.002)**	**-1.195 (-1.942 to -0.449)**	-0.273 (-0.780 to 0.234)	0.415 (-0.151 to 0.981)
**Fasting glucose (mmol/L)**						
Average acceleration (m*g*)	**-0.076 (-0.150 to -0.001)**	0.013 (-0.046 to 0.071)	0.050 (-0.023 to 0.122)	-0.060 (-0.143 to 0.023)	-0.022 (-0.093 to 0.050)	-0.015 (-0.102 to 0.073)

	Axivity			ActivPAL		
**Health marker**	**Unadjusted**	**Model 1**	**Model 2**	**Unadjusted**	**Model 1**	**Model 2**
Intensity gradient	**-0.096 (-0.172 to -0.019)**	-0.040 (-0.105 to 0.024)	-0.068 (-0.148 to 0.011)	-0.006 (-0.070 to 0.059)	-0.021 (-0.082 to 0.040)	-0.013 (-0.089 to 0.062)
**HbA1c (%)**						
Average acceleration (m*g*)	**-0.086 (-0.123 to -0.048)**	**-0.037 (-0.066 to -0.009)**	**-0.025 (-0.062 to 0.012)**	**-0.059 (-0.097 to -0.020)**	**-0.040 (-0.071 to -0.009)**	-0.036 (-0.077 to 0.005)
Intensity gradient	**-0.070 (-0.109 to -0.031)**	**-0.037 (-0.067 to -0.007)**	**-0.023 (-0.062 to 0.016)**	-0.025 (-0.063 to 0.014)	-0.027 (-0.059 to 0.005)	-0.008 (-0.049 to 0.034)
**Triglycerides (mmol/L)**						
Average acceleration (m*g*)	**-0.066 (-0.109 to -0.022)**	-0.035 (-0.078 to 0.008)	-0.001 (-0.056 to 0.054)	**-0.091 (-0.140 to -0.042)**	**-0.089 (-0.141 to -0.037)**	**-0.105 (-0.162 to -0.048)**
Intensity gradient	**-0.088 (-0.134 to -0.042)**	**-0.064 (-0.110 to -0.019)**	**-0.064 (-0.122 to -0.006)**	-0.015 (-0.061 to 0.030)	-0.024 (-0.073 to 0.024)	0.030 (-0.021 to 0.082)
**HDL cholesterol**						
Average acceleration (m*g*)	**0.045 (0.012 to 0.078)**	**0.049 (0.018 to 0.080)**	0.033 (-0.004 to 0.070)	**0.035 (0.001 to 0.069)**	**0.066 (0.033 to 0.099)**	**0.067 (0.027 to 0.107)**
Intensity gradient	0.024 (-0.007 to 0.054)	**0.049 (0.019 to 0.079)**	0.03 (-0.006 to 0.065)	0.021 (-0.011 to 0.053)	**0.033 (0.003 to 0.063)**	-0.002 (-0.038 to 0.034)
**LDL cholesterol**						
Average acceleration (m*g*)	-0.018 (-0.152 to 0.117)	-0.012 (-0.152 to 0.128)	0.011 (-0.139 to 0.162)	0.017 (-0.111 to 0.144)	-0.009 (-0.150 to 0.132)	-0.039 (-0.196 to 0.117)
Intensity gradient	-0.045 (-0.141 to 0.052)	-0.035 (-0.140 to 0.070)	-0.042 (-0.139 to 0.056)	0.083 (-0.010 to 0.177)	0.034 (-0.065 to 0.134)	0.055 (-0.047 to 0.158)
**Total cholesterol**						
Average acceleration (m*g*)	**-0.102 (-0.184 to -0.020)**	-0.083 (-0.165 to 0.000)	-0.077 (-0.173 to 0.018)	-0.080 (-0.171 to 0.011)	-0.076 (-0.171 to 0.018)	**-0.122 (-0.227 to -0.018)**

	Axivity			ActivPAL		
**Health marker**	**Unadjusted**	**Model 1**	**Model 2**	**Unadjusted**	**Model 1**	**Model 2**
Intensity gradient	**-0.106 (-0.189 to -0.024)**	-0.053 (-0.138 to 0.031)	-0.010 (-0.107 to 0.088)	0.061 (-0.027 to 0.149)	0.023 (-0.065 to 0.111)	0.087 (-0.010 to 0.183)
**Systolic blood pressure**						
Average acceleration (m*g*)	-1.296 (-2.713 to 0.121)	-0.658 (-1.924 to 0.608)	0.055 (-1.425 to 1.535)	-0.505 (-1.721 to 0.711)	**-1.384 (-2.482 to -0.286)**	**-1.949 (-3.316 to -0.582)**
Intensity gradient	**-1.430 (-2.811 to -0.048)**	**-1.295 (-2.530 to -0.059)**	-1.326 (-2.776 to 0.124)	**1.381 (0.036 to 2.726)**	0.053 (-1.201 to 1.307)	1.065 (-0.446 to 2.576)
**Diastolic blood pressure**						
Average acceleration (m*g*)	**-1.698 (-2.493 to -0.903)**	**-1.314 (-2.073 to -0.555)**	-0.911 (-1.828 to 0.006)	**-0.869 (-1.640 to -0.097)**	**-1.147 (-1.886 to -0.408)**	**-1.640 (-2.569 to -0.711)**
Intensity gradient	**-1.481 (-2.301 to -0.660)**	**-1.261 (-2.070 to -0.452)**	-0.749 (-1.728 to 0.230)	0.665 (-0.173 to 1.502)	0.078 (-0.743 to 0.899)	0.930 (-0.068 to 1.927)
**Mean arterial blood pressure**						
Average acceleration (m*g*)	**-1.564 (-2.516 to -0.612)**	**-1.095 (-1.967 to -0.223)**	-0.589 (-1.610 to 0.431)	-0.748 (-1.618 to 0.123)	**-1.226 (-2.027 to -0.425)**	**-1.743 (-2.749 to -0.737)**
Intensity gradient	**-1.464 (-2.429 to -0.499)**	**-1.272 (-2.173 to -0.371)**	-0.941 (-2.002 to 0.120)	0.903 (-0.066 to 1.873)	0.070 (-0.853 to 0.992)	0.975 (-0.140 to 2.089)

All values are expressed as coefficient (95% CI)

Bold numbers indicate significant regression coefficient ($p < 0.05$).

Model 1 adjusted for potential covariates – age, sex, ethnicity, smoking status, medical history of type 2 diabetes, lipid lowering medication or beta blockers and deprivation score.

Model 2 included volume and intensity into the model.

Supplementary Table S2. MX values: M720. M480, M240, M120, M60, M30, M15, M5 and M2 for the activPAL and Axivity devices.

Metric	ActivPAL	Axivity
M16h	3.1 (2.9 - 3.3)	3.4 (3.3 - 3.5)
M12h	7.3 (7 - 7.6)	7.6 (7.3 - 7.9)
M10h	9.5 (9.2 - 9.8)	11.4 (11 - 11.8)
M8h	12.2 (11.9 - 12.5)	17.8 (17.2 - 18.4)
M6h	16.1 (15.7 - 16.5)	29 (28.2 - 29.8)
M240	24.4 (23.7 - 25.1)	48.6 (47.5 - 49.7)
M120	61.1 (58.7 - 63.5)	87.9 (86.3 - 89.5)
M60	143.6 (138.7 - 148.5)	134.6 (131.5 - 137.7)
M30	228 (222.2 - 233.8)	189.6 (183.9 - 195.3)
M20	266 (259.9 - 272.1)	225.9 (218.3 - 233.5)
M15	287.2 (281 - 293.4)	249.7 (240.8 - 258.6)
M10	310.9 (304.7 - 317.1)	282.4 (272.3 - 292.5)
M5	341.5 (335.3 - 347.7)	339.5 (327.9 - 351.1)
M2	372.7 (366.3 - 379.1)	426.5 (412.4 - 440.6)
M1	393.3 (386.6 - 400)	503.5 (487 - 520)

Mean MX values are expressed as milligravitational units. MX, most active X minutes.

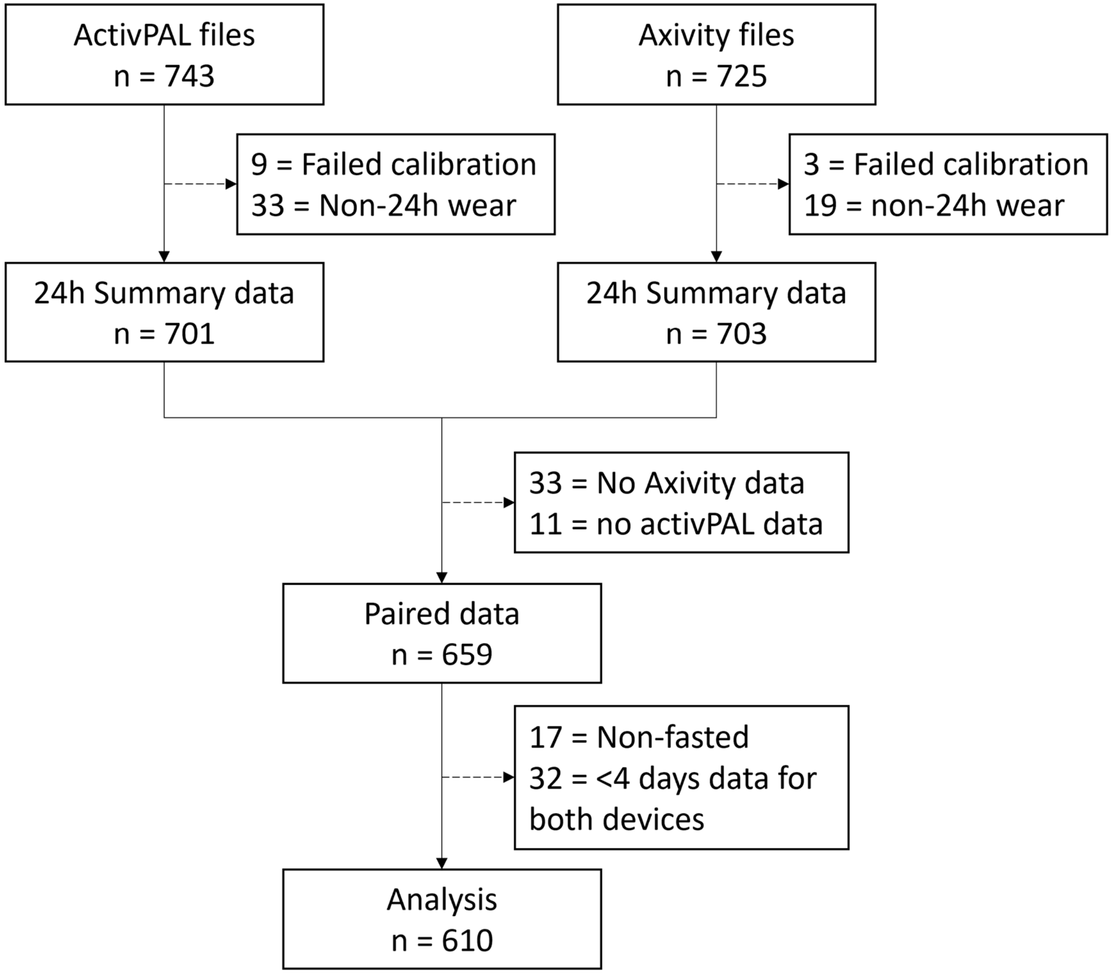

Supplementary Figure S1. Flow of participants through the study.
